# Application of RSM for optimization of glutamic acid production by *Corynebacterium glutamicum* in bath culture

**DOI:** 10.1016/j.heliyon.2021.e07359

**Published:** 2021-06-24

**Authors:** Azadeh Fahimitabar, Seyyed Mohammad Hossein Razavian, Seyyed Ali Rezaei

**Affiliations:** Department of Microbiology, Qom Branch, Islamic Azad University, Qom, Iran

**Keywords:** *C. glutamicum*, Culture optimization, Glutamic acid, Response surface methodology

## Abstract

Glutamate plays an important role in different cellular processes. Its new applications in various industries have led to an increase in the production of it while fermentation is a very important economically method. In this study, the production of glutamate by the wild type of *Corynebacterium glutamicum* PTCC(Persian Type Culture Collection) 1532 was optimized using RSM.

Central Composite Design (CCD) was developed by Design-Expert software version 12.0.3.0 (dx-12, State-Ease Inc.) to evaluate the effect of four important variables in five levels on glutamate production. TLC was employed to evaluate glutamate in medium qualitatively and then quantitative estimation was done by HPLC.

Normal probability analysis demonstrated that data has a normal distribution. The results of ANOVA analysis showed that the urea concentration both alone and with temperature is the most effective variable in the fermentation process. Based on the quadratic model obtained in CCD, temperature 30 °C; glucose 9 g.dL^−1^; biotin 9 μg.L^−1^ and urea concentration of 0.3 g.dL^−1^ were found optimum conditions with a predicted glutamate production of 19.84 mg.mL^−1^ with desirable level 1.

Therefore RSM can be an effective method to optimize glutamate production and the findings of this study are a guideline for the other amino acids fermentation by *C*. *glutamicum*.

## 1 Introduction

Glutamic acid (Glu) was discovered in 1866 by the German chemist Karl Heinrich Ritthausenas. Glu has a linear backbone with two carboxyl groups and a net negative charge at physiological pH and is one of the most abundant amino acids in the human body [1]. Glu not only acts as the precursor to other amino acids and biological molecules (glutathione, adrenalin, acetyl choline) but plays an important role in cellular processes such as intracellular signaling, nitrogen metabolism, energy production, intracellular signaling and cell wall synthesis [2]. Its highest concentrations are present in the brain and muscles and it is needed on a daily basis to survive [3]. .

The body uses glutamate to produce GABA (γ-amino butyric acid), an inhibitory neurotransmitter that participate in learning and muscle contraction. So, Glu is used to cure personality disorders in children, muscular dystrophy, epilepsy, Parkinson, Schizophrenia, alcoholism, the healing of ulcers and improving mental capacities [4, 5]. On the other hand, glutamate receptors are present on immune cells (T cells, B cells, macrophages, and dendritic cells), which suggests that glutamate plays a role in both the innate and adaptive immune system [6]. Glu can be safely used in pharmaceutical applications for improvement of intestine function [4], as a dietary supplement for patients and elderly people with malnutrition, in animal nutrition and making artificial leather [7].

Glutamate can be prepared by variety methods but fermentation is the most economical way and many efforts were being made to reduce the cost of glutamate production [8, 9]. In 1957, a glutamate producing bacterium was discovered and named *Micrococcus glutamicus* which now a days called *Corynebacterium glutamicum* (*C. glutamicum*) [10]. It is a rod shaped, gram positive, non-mobile, positive catalase, non-pathogenic bacteria with metachromatic granules and without sporulation. This bacterium is often aerobic, auxotroph biotin and grows very fast. The ability of this strain to consume a wide range of carbon sources like glucose, alcohols and organic acids is the most important reason for its choice to produce glutamate [11, 12].

Many researches by routine one factor at a time method have been done to optimize the production of Glu by different bacterium [[13], [14], [15]]. But these methods involve changing one independent parameter simultaneously with stabilizing other parameters (Taguchi method). Taguchi one-dimensional method is simple but laborious, time-consuming and often unable to reach optimum conditions because it does not consider the interaction of factors. Overcome these problems, the optimization by RSM is an effective strategy to access optimum conditions in multiple variable systems. It used mathematical and statistical methods to find a combination of independent variables to access optimum conditions by study the effect of variables on the response at a time in the RSM [16].

RSM, in spite of possible errors, can change complex problems into a simple problem with high accuracy and also determine response sensitivity to each variable [17]. One of the advantages is to reduce the number of tests, save time and no need for complex measurements to analyze data. It allows to study the relationship between variables and also to determine the most appropriate conditions and predictable results [16]. In this study, a number of parameters were used to increase the production of glutamate by *C. glutamicum* PTCC 1532 in fermentation were investigated and optimized by using statistical response surface methodology (RSM).

## 2 Materials and methods

### 2.1 Microorganism, culture media and chemicals

All materials used in this study, such as glucose, ammonium sulfate, methionine and glutamic acid (to draw the standard curve) were purchased from Merck Company (Germany). All media and chemicals were used without any pretreatment. The bacterium used in this study was *Corynebacterium glutamicum* strain PTCC(Persian Type Culture Collection) 1532 and was prepared from the regional center of the Iranian Industrial Bacteria Collection (PTCC) as a lyophilized packed ampoule.

### 2.2 Activation of bacteria and inoculum preparation

**Figure 1:**
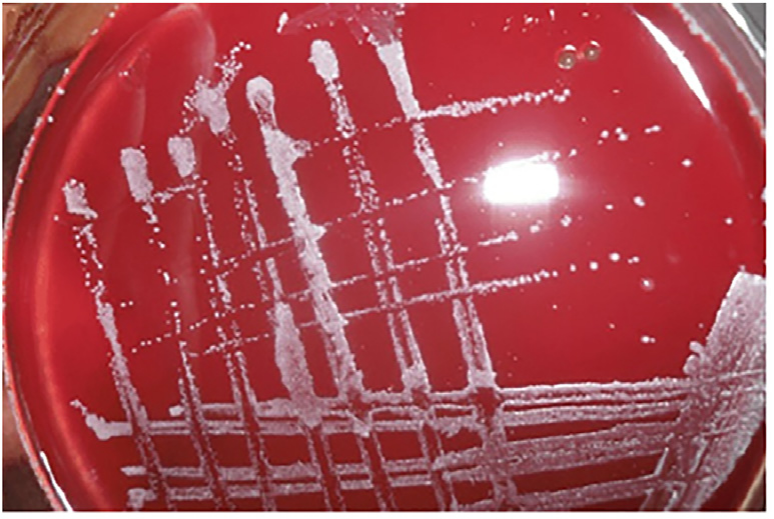
*Corynebacterium glutamicum* strain PTCC 1532 cultured on blood agar medium at 37 °C.

For activating, the bacterium was cultured for 48 h at 37 °C on blood agar medium (Figure 1). After the appearance of yellow colonies, they identified by microbial and biochemical tests and transferred to 4 °C. Inoculum was prepared by transferring a loopful of colonies to 250 ml Erlenmeyer flask containing Minimal salt medium (MSM): glucose 5, yeast extract 0.5, MgSO_4_.7H_2_O 0.2, FeSO_4_.7H_2_O 0.001, MnSO_4_,H_2_O 0.001, KH_2_PO_4_ 0.1, K_2_HPO_4_ 0.1 (all of them g.dL^−1^), Biotin 20 Pg.L^−1^ [18].

### 2.3 Fermentation medium

The medium composition for the production of glutamate consist of: MgSO_4_.7H_2_O 0.2, FeSO_4_.7H_2_O 0.001, MnSO_4_.H_2_O 0.001, KH_2_PO_4_ 0.1, K_2_HPO_4_ 0.1 (all of them g.dL^−1^). The medium pH was adjusted to 7.0 with sodium hydroxide or hydrochloric acid. The fermentation was carried out in a 100 ml Erlenmeyer flask containing 50 ml of medium. The fermentation medium was inoculated with 10 ml of the overnight culture. The production medium was kept in an orbital shaker at 180 rpm with designed temperatures for 48 h. After 20 h of incubation time, 4 U/mL penicillin G was added to releasing cellular glutamate into the medium.

### 2.4 Design of experiments

#### 2.4.1 Central Composite Design (CCD)

CCD is a statistical method based on the multivariate nonlinear model for the optimization of variables in a process. In the present study, the CCD was applied to study the interactions of the various factors affecting the amino acid fermentation and to determine the optimum condition for glutamate production using *C.glutamicum*. The design of experiment and statistical analysis performed using Design-Expert software version 12.0.3.0 (dx-12, State-Ease Inc). Four variable factors (X_1_: temperature, X_2_: glucose concentration, X_3_: biotin concentration, X_4_: urea concentration) assigned to five levels including: high level (+1), low level (-1), medium level (central point) (0) and 2 axial points (±α) as shown in Table 1. The CCD consisted of 27 experimental runs (16 factorial points, 8 axial points, 3 central points) as shown in Table 2. The results showed *C. glutamicum* was able to produce glutamate under all the considered conditions.

**Table 1:** Experimental variables and their coded levels for the central composite design.

Independent variable	Unit	Symbol Code	Levels of coded variables				
			- α -2	Low -1	Medium 0	High +1	+ α +2
Temperature	^°^C	X_1_	25	30	35	40	45
Glucose	g.dL^−1^	X_2_	3	6	9	12	15
Biotin	μg.L^−1^	X_3_	0	3	6	9	12
Urea	g.dL^−1^	X_4_	0.1	0.3	0.5	0.7	0.9

**Table 2:** Experimental factors in coded and actual units and experimental responses.

Run	Independent variable in coded form				Independent variable in actual form				Glutamic acid (mg.mL^−1^)	
	X_1_	X_2_	X_3_	X_4_	X_1_ (^°^C)	X_2_ (g.dL^−1^)	X_3_ (μg.L^−1^)	X_4_ (g.dL^−1^)	Actual	Predicted
1	-1	1	1	1	30	12	9	0.7	3.1	2.95
2	0	0	2	0	35	9	12	0.5	14.9	14.94
3	0	0	0	0	35	9	6	0.5	17.3	17.57
4	1	1	-1	-1	40	12	3	0.3	11.68	11.46
5	-1	-1	1	-1	30	9	9	0.3	19.9	19.84
6	-1	1	-1	1	30	12	3	0.7	3.8	3.67
7	1	-1	-1	-1	40	6	3	0.3	8.3	8.39
8	-1	-1	1	1	30	6	9	0.7	8.7	8.78
9	1	1	-1	1	40	12	3	0.7	9	9.09
10	0	0	0	2	35	9	6	0.9	2.78	3.01
11	2	0	0	0	45	9	6	0.5	6.65	6.75
12	0	0	0	-2	35	9	6	0.1	16.4	16.36
13	-1	-1	-1	1	30	6	3	0.7	8.7	8.57
14	0	0	-2	0	35	9	0	0.5	13	13.16
15	1	-1	1	1	40	6	9	0.7	8.1	8.11
16	1	1	1	1	40	12	9	0.7	8.28	8.08
17	1	-1	-1	1	40	6	3	0.7	8.4	8.18
18	-1	1	-1	-1	30	12	3	0.3	14.3	14.23
19	0	2	0	0	35	15	6	0.5	8.3	8.47
20	1	-1	1	-1	40	6	9	0.3	10.9	10.89
21	1	1	1	-1	40	12	9	0.3	12.96	13.03
22	-1	1	1	-1	30	12	9	0.3	16	16.08
23	0	0	0	0	35	9	6	0.5	17.5	17.57
24	0	0	0	0	35	9	6	0.5	17.9	17.57
25	-2	0	0	0	25	9	6	0.5	10.1	10.20
26	0	-2	0	0	35	3	6	0.5	11.2	11.23
27	-1	-1	-1	-1	30	6	3	0.3	16.9	16.97

### 2.5 Extraction of intracellular glutamate

The fermentation broth was centrifuged at 8000 g for 15 min and the supernatant used for glutamate estimation. The cells and debris diluted by citric acid 7 % and kept in -80 °C for the next analysis.

### 2.6 Analytical methods

#### 2.6.1 Thin layer chromatography (TLC) analysis

Silica gel ascending TLC employed for estimating glutamate in the culture medium with mobile phase n-butanol: acetic acid: water (1:2:4 v/v). Spots appeared by 0.5 % ninhydrin in n-butanol solution spraying and heating.

#### 2.6.2 Colorimetric assay

**Figure 2:**
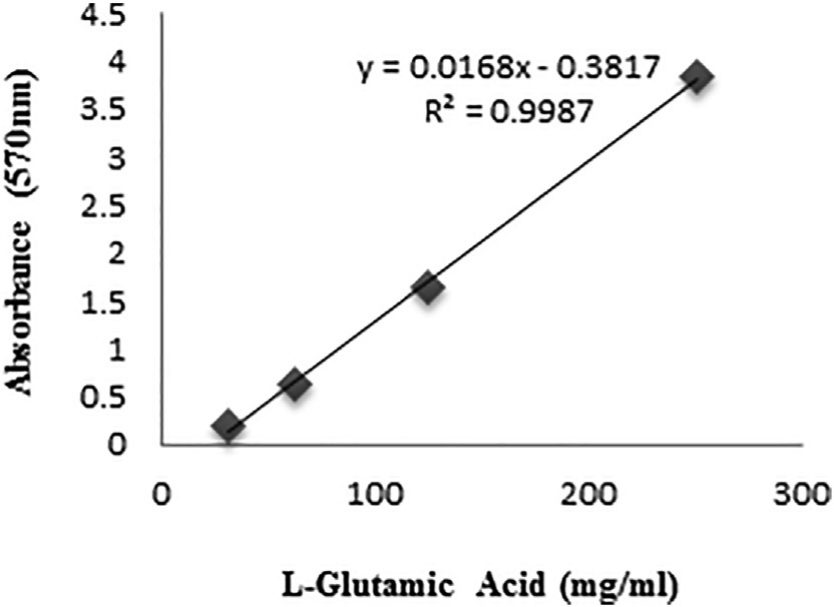
Standard curve of Glutamate concentration.

The quantitative evaluation of glutamate using the ninhydrin solution was done based on Chinard colorimetric method [19]. Chinard reagent (C) was prepared by dissolving 25 mg ninhydrin in 10 ml solvent solution (containing 4 ml of 6 M sulfuric acid and 6 ml of glacial acetic acid). 1ml of culture media was mixed to 1 ml of C reagent and the samples were placed in a boiling water bath for one hour. Then, 1 ml of acetic acid was added and the absorbance was measured at 570 nm using a UV-V is spectrophotometer. The color intensity adjusted to glutamate standard concentrations. Standard curve “absorbance vs. glutamate concentration” was drawn by different concentrations of glutamic acid. Glutamate concentrations in supernatants were estimated using linear regression analysis equation derived from standard curve (Figure 2).

#### 2.6.3 HPLC analysis

The glutamate concentration determined exactly based on Yang et al method by reversed phase high-performance liquid chromatography (RP-HPLC. HPLC system equipped with a C18 column (250 × 4.6 mm) and UV detector. Mobile phase consisted of 60 % solution A (aqueous solution of 10.254 g sodium acetate, 0.5 ml tri-ethylamine and 0.7 ml acetic acid in 1000 ml, pH 5.8), 12 % solution B (acetonitrile) and 28 % solution C (deionized water) mixture. The flow rate and the injection volume were 0.6 ml/min and 20 μl respectively. Amino acid precolumn derivatization was done by Phenyl iso thio cyanate (PITC) while produced Phenyl iso thio carbamate-glutamate (PITC-glutamate) had UV absorbance at 254 nm [20].

## 3 Results

### 3.1 Model fitting and statistical analysis

The effects of four independent variables on glutamic acid production were analyzed using CCD. The results of the glutamate production as measured by HPLC and predicted values were summarized in Table 2. The analysis of variance (ANOVA) and regression coefficients for the resulting model are presented in Table 3.

**Table 3:** Analysis of variance (ANOVA) for response surface quadratic model for Glutamic Acid production.

Source	Sum of Squares	Df	Mean Square	F-value	p-value	
Model	582.49	14	41.61	866.61	<0.0001	Significant
X_1_ (temperature)	17.82	1	17.82	371.15	<0.0001	
X_2_ (glucose)	11.45	1	11.45	238.57	<0.0001	
X_3_ (biotin)	4.73	1	4.73	98.62	<0.0001	
X_4_ (urea)	267.33	1	267.33	5568.24	<0.0001	
X_1_.X_2_	33.70	1	33.70	701.89	<0.0001	
X_1_.X_3_	0.0812	1	0.0812	1.69	0.2178	
X_1_.X_4_	66.99	1	66.99	1395.41	<0.0001	
X_2_.X_3_	0.8742	1	0.8742	18.21	0.0011	
X_2_.X_4_	4.69	1	4.69	97.63	<0.0001	
X_3_.X_4_	6.63	1	6.63	138.11	<0.0001	
X_1_^2^	110.25	1	110.25	2296.41	<0.0001	
X_2_^2^	79.43	1	79.43	1654.44	<0.0001	
X_3_^2^	16.50	1	16.50	343.78	<0.0001	
X_4_^2^	82.76	1	82.76	1723.74	<0.0001	
Residual	0.5761	12	0.0480			
Lack of Fit	0.3895	10	0.0389	0.4173	0.8588	not significant
Pure Error	0.1867	2	0.0933			
Correlation Total	583.06	26				

In the Table 3, degree of freedom, sum of squares, mean squares, significant level (P-value) and Fisher test (F-value) are presented. The P-value serves as a tool for checking the significance of each term [16]. The model had a very low P-value (P < 0.0001), which implied that the model fitted the experimental data significantly. F value indicates the effect of different variables on the fermentation process of glutamate as following:

**Urea concentration > Temperature > Glucose concentration > Biotin concentration**

So urea concentration (F = 5568) is the most effective factor in the fermentation process. The obtained results indicated that the main variables and interaction effects of all variables except temperature and Biotin concentration (X_1_, X_3_) are significant (P0.05). The significant levels play an important role to determine the significance of the interaction effect of variables. Whatever P value is low, the model is more valid. The lack of fit to pure error is not significant in this model. In statistical models, lack of fit for pure errors should not be significant, that is, it should be 0.05 % to confirm the model and indicating the validity of the response surface results. According to values (P value ≤0.05) and (F value = 866), it is determined that proposed model is significant. The value 98 % is related to (R^2^ adj). The R squared value was used to describe the variability in the actual response values that could be explained by the experimental factors and their interactions [16]. The precision adequacy (102.908) shows the power of chosen model at confident level 95 % showing the power of model is high. The changes coefficient is 1.94 % showing low changes of chosen model around standard deviation and also it indicates the reliability of the model.

### 3.2 Validation of the model

Validation of the statistical model and regression equation was conducted by temperature 30 °C, glucose 9g.dL^−1^, biotin 9μg.L^−1^ and urea concentration 0.3 g.dL^−1^. Under these optimized conditions, the predicted response for glutamate production was 19.84 mg/ml. These results confirmed the validity of the model and the experimental values were quite close to the predicted values.

**Figure 3:**
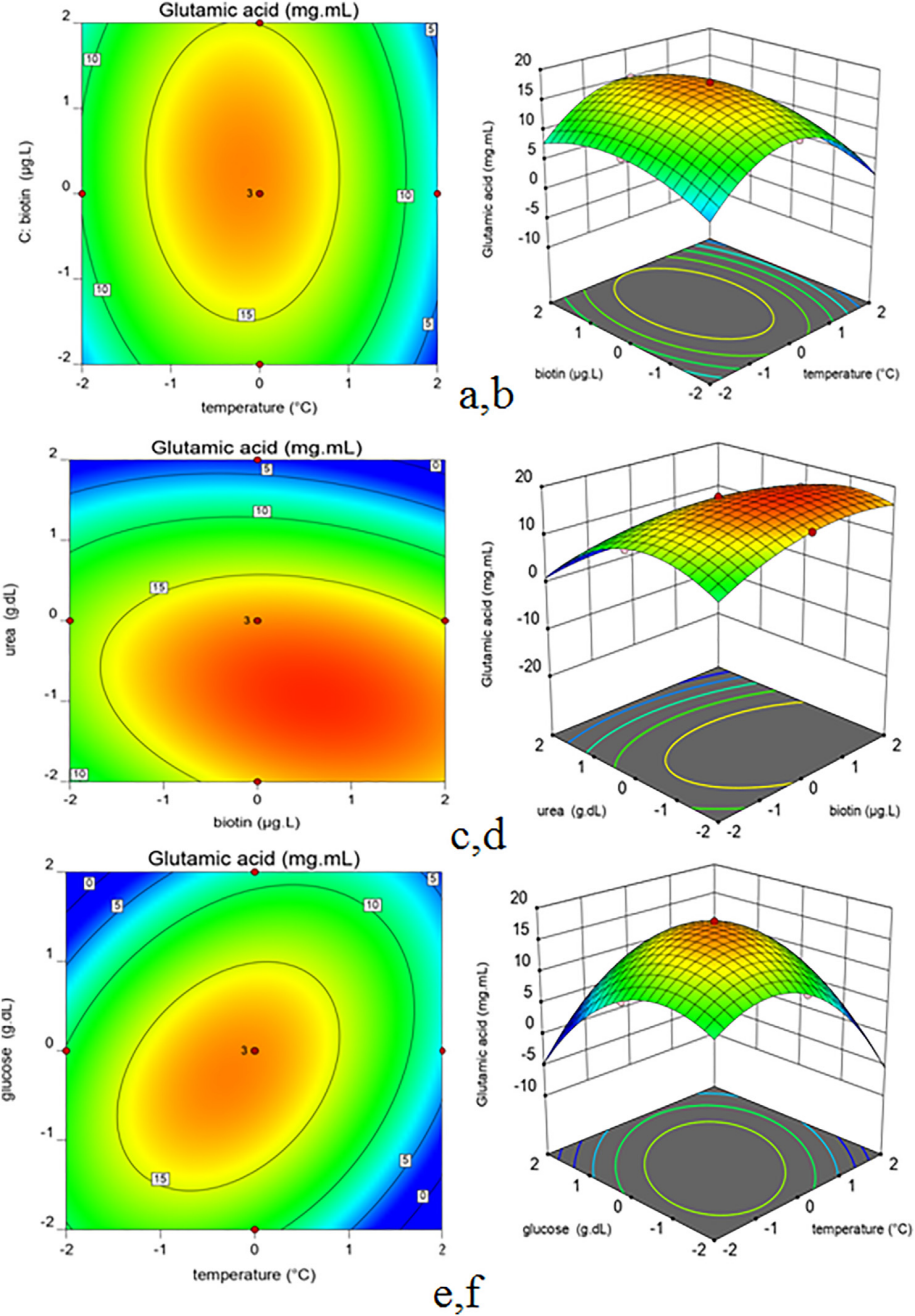
Surface and contour plots of the effects off our factors on production of Glutamate. a, b: Interaction of X_1_ (Temperature) and X_3_ (Biotin); c, d: Interaction of X_4_ (Urea) and X_3_ (Biotin); e, f: Interaction of X_1_ (Temperature) and X_2_ (Glucose).

**Figure 4:**
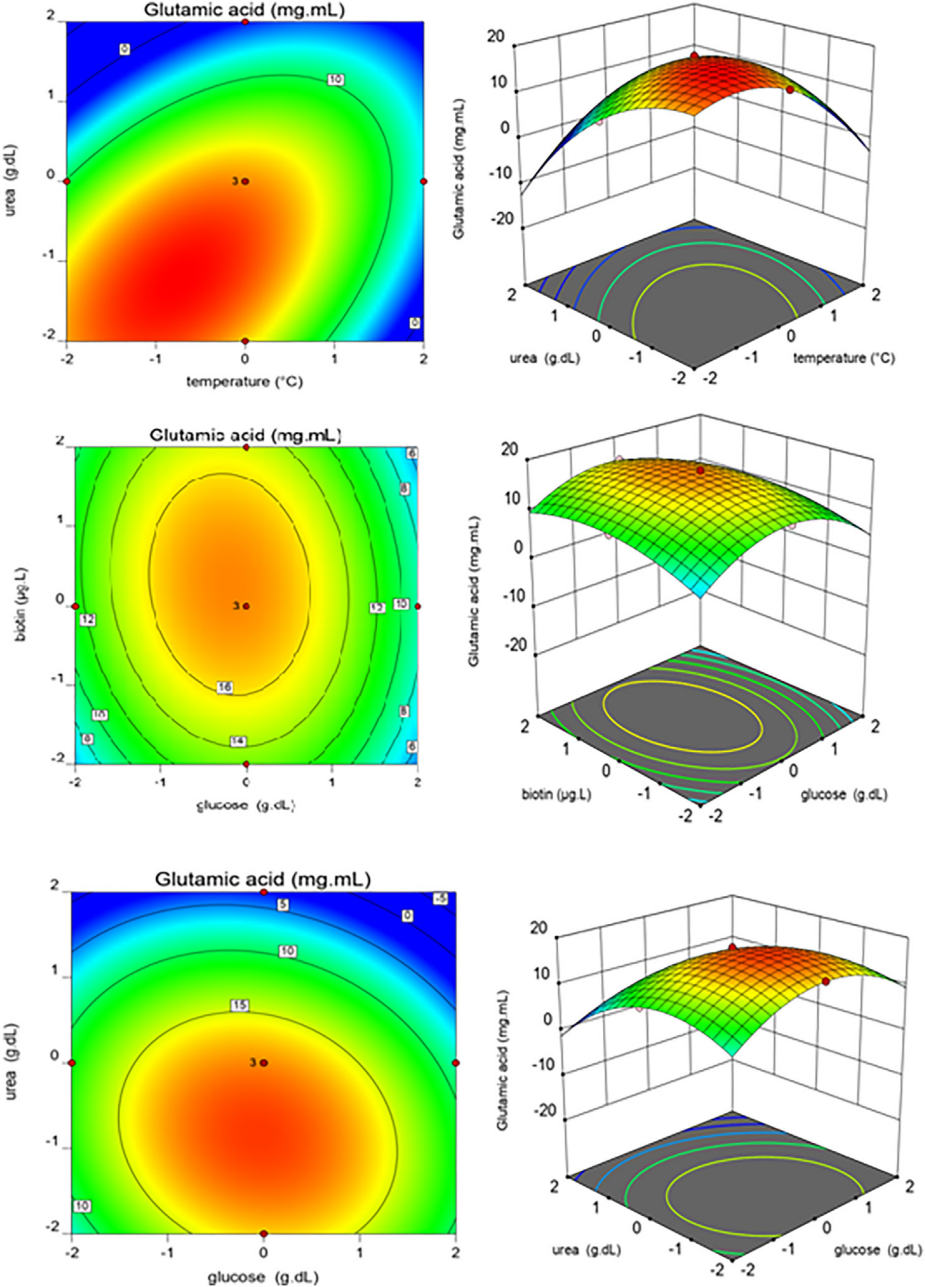
Surface and contour plots of the effects of four factors on production of Glutamate. a, b: Interaction of X_1_ (Temperature) and X_4_ (Urea); c, d: Interaction of X_2_ (Glucose) and X_3_ (Biotin); e, f: Interaction of X_2_ (Glucose) and X_4_ (Urea).

The next step was to obtain the optimum value for each factor to get the maximum response. The 3D response surface plots and the 2D contour plots that could evaluate the interactive effects of the two factors on the response were in Figure 3 and Figure 4. Graphical diagrams show the effect of four variables (temperature X_1_, glucose concentration X_2_, Biotin concentration X_3_ and urea concentration X_4_) on the production of glutamate generated by the predicted model. The diagrams are indicated as functions of two variables on the maximal glutamate production, while the other two variables were held at the center point.

## 4 Discussion

Glutamate is one of the non-essential amino acids and a constituent of almost all proteins. The new and diverse applications of glutamate and its salt in various medical, nutritional and industrial fields have created an increasing need for it [3]. Glutamate can be prepared by various methods such as extraction from hydrolysis of proteins, chemical synthesis, protoplast fusion technique and recombinant DNA technology but microbial fermentation is still the most economical way [8, 9]. In this context, three fields of studies are conducting:

- finding new strains and/or improving the performance of microbial strains
- optimizing culture conditions and compounds of fermentation media
- improving separation and purification techniques.

*C. glutamicum* is a Gram positive, facultative anaerobic, non-spore forming bacterium that generally recognized as a safe and good candidate for the fermentation process in this field [21].

One factor at a time (Taguchi) method is the common way to access desirable results in fermentation and has been used for the culture optimization of different bacteria in a lot of researches [15, 22, 23, 24, 25, 26, 27]. In this simple and single-dimensional method, all factors influencing results are constant and every time, only one factor is changed and its effect on response is studied. Among the disadvantages of this method is failure to review of the interaction of variables, no access to optimum conditions and increasing of time, cost and consuming chemical materials. Overcome these problems, the optimization by RSM is an effective strategy to access optimum conditions in multiple variable systems. RSM reduces the number of tests, save time and need for complex measurements to analyze data [16] and used in a lot of researches [28, 29, 30, 31]. RSM successfully have been used to optimize fermentation of *C. glutamicum* to improve different metabolites [26, 32, 33] and Glu production [34, 35, 36].

The present study aimed to improve the glutamate production by *C. glutamicum* PTCC 1532 in the fermentation process by using statistical RSM. We prepared a base medium and tried to optimize the fermentation of glutamate by changing many key factors affecting on the Glutamate production based on literatures. Then, using these factors, glutamate production was optimized by the RSM.

**Glucose concentration:** Many published articles have suggested the use of different sources of glucose can stimulate *C. glutamicum* growth and induce metabolic processes [37, 38, 39, 40] and Naiyf S. Alharbi rt al. (2020) Showed that pure glucose has the highest efficiency of glutamic acid production by *C. glutamicum13032* [34]. **Temperature**: Previously Lapujade et al. (1999) were obtained the highest cell growth rate at 33 °C and the highest extracellular secretion of glutamate at 40 °C. They observed a six-fold increase in the production of glutamate due to increasing temperature [41]. **Biotin**: Since biotin is a cofactor of acetyl-CoA carboxylase, which is necessary for fatty acid synthesis, it is thought that the cell membrane permeability increased when biotin was decreased in the culture medium. Shiio et al. 1962 showed the production of glutamate was significant when biotin is depleted [42]. **Ammonia**: On the other hand, glutamate is synthesized from 2-oxogluratrate by a one-step reaction catalyzed by glutamate dehydrogenase (GDH) which is the main pathway for glutamate formation when the ammonium concentration is sufficiently high [43]. Therefore urea, glucose and biotin concentration in addition to temperature as key factors have a specific effect on glutamate production selected and optimized.

Analysis of variance (ANOVA) showed the significance and the validity of the model which confirmed by the verification experiments [44]. Results indicated the urea, glucose, biotin concentration and temperature are respectively the most effective variables on the fermentation process of glutamate. Also the obtained results indicated that urea concentration (F = 5568) and combined of temperature/urea (F = 1395.4) have the most extensive effects on fermentation process (Table3).

The highest Glu production was observed at run 5 (19.9 mg.ml^−1^) and the lowest at run 10 (2.78 mg.ml^−1^). The optimal conditions were evaluated as follows: temperature 30 °C, glucose concentration 9 g.dL^−1^, biotin concentration 9 μg.L^−1^, urea concentration 0.3 g.dL^−1^.

## 5 Conclusion

Findings showed that the CCD could be applied for evaluating of the variables influencing on glutamate fermentation and determining the mentioned process optimization condition.The optimal conditions were evaluated as: temperature 30 °C, glucose concentration 9 g.dL^−1^, biotin concentration 9 μg.L^−1^, urea concentration 0.3 g.dL^−1^. The highest glutamate production was 19.9 mg.ml^−1^.

## Declarations

### Author contribution statement

Azadeh Fahimitabar: Performed the experiments; Analyzed and interpreted the data; Wrote the paper.

Seyyed Mohammad Hossein Razavian: Conceived and designed the experiments; Analyzed and interpreted the data; Contributed reagents, materials, analysis tools or data; Wrote the paper.

Seyyed Ali Rezaei: Analyzed and interpreted the data; Contributed reagents, materials, analysis tools or data.

### Funding statement

This research did not receive any specific grant from funding agencies in the public, commercial, or not-for-profit sectors.

### Data availability statement

No data was used for the research described in the article.

### Competing interest statement

The authors declare no conflict of interest.

### Additional information

No additional information is available for this paper.
